# Effects of steroid hormones on differentiated glandular epithelial and stromal cells in a three dimensional cell culture model of the canine endometrium

**DOI:** 10.1186/1746-6148-9-86

**Published:** 2013-04-24

**Authors:** Cordula Bartel, Alexander Tichy, Susanne Schoenkypl, Christine Aurich, Ingrid Walter

**Affiliations:** 1 Department of Pathobiology, Institute of Anatomy, Histology and Embryology, University of Veterinary Medicine, Veterinaerplatz 1, Vienna A – 1210, Austria; 2 Department of Biomedical Science, Institute of Population Genetics, Platform Biostatistics, University of Veterinary Medicine, Veterinaerplatz 1, Vienna, A – 1210, Austria; 3 Small Animal Practice, Vienna, 1220, Austria; 4 Centre for Artificial Insemination and Embryo Transfer, University of Veterinary Medicine, Veterinaerplatz 1, Vienna, A – 1210, Austria; 5 VetCore Facility for Research, University of Veterinary Medicine, Veterinaerplatz 1, Vienna, A – 1210, Austria

**Keywords:** Dog, Endometrium, 3D cell culture, Glandular epithelium, Stroma, Steroid hormones, Oestrogen receptor, Progesterone receptor, Proliferative activity, Immunohistochemistry, Apical polarity

## Abstract

**Background:**

Oestrogens and progesterone have a significant impact on the endometrium during the canine oestrous cycle. Their receptors mediate plasma steroid hormone levels and are expressed in several endometrial cell types. Altered steroid receptor expression patterns are involved in serious uterine diseases; however the mechanisms of hormone action during pathogenesis in these tissues remain unclear. The development of 3D culture systems of canine endometrial cells provides an opportunity for the effects of steroid hormones to be quantitatively assessed in a more *in vivo*-like setting. The present study aimed to determine the effects of the steroid hormones 17β-estradiol (E) and progesterone (P) on the expression of the oestrogen and progesterone receptors (ER and PR), and on proliferative activity, in a 3D co-culture system of canine uterine origin, comprising differentiated endometrial glands, and stromal cells (SCs).

**Results:**

Morphology, differentiation, and apical-basolateral polarity of cultured glandular epithelial cells (GECs) were comparable to those in native uterine tissue as assessed by immunohistochemistry using differentiation markers (β-catenin, laminin), lectin histochemistry, and transmission electron microscopy. Supplementation of our 3D-culture system with E (at 15, 30 and 100 pg/mL) resulted in constant levels of ER expression in GECs, but reduced expression levels in SCs. PR expression was reduced in both GECs and SCs following treatment with E. 3 ng/mL P resulted in increased ER expression in GECs, but a decrease in SCs. PR expression in GECs increased in all P-treated groups, whereas PRs in SCs decreased with the lowest and highest doses, but increased with the middle dose of treatment. Proliferative activity, assessed by Ki67 staining, remained below 1% in all assays and cell types.

**Conclusions:**

The present study demonstrates the applicability of our 3D organotypic canine endometrium-derived culture system for cellular-level studies. 3D cultures represent near-physiological systems allowing reproducible quantitative experimentation, thus reducing the need to experiment on living animals. The results of the present investigation emphasize the importance of co-culture of the uterine glands with SCs, as it was shown that the responsiveness of the different cell types to steroid hormones were divergent in the 3D cell culture model.

## Background

Altered patterns of oestrogen receptor (ER) and progesterone receptor (PR) expression have been suggested to play roles in the etiology of serious, occasionally life-threatening pathological alterations of the canine endometrium, concerning mainly the uterine surface, glands and the stroma, including cystic endometrial hyperplasia (CEH), and pyometra [1]. Alterations in the plasma levels of progesterone are involved [2,3], but detailed knowledge of processes controlling these serious endometrial alterations at the cellular and molecular levels is lacking. As a prerequisite tool to study the effects of steroid hormones and other processes on the cellular level, a three dimensional (3D) co-culture system of the canine endometrium was established in our laboratory [4], and further developed for the present study. Previous *in vitro* studies demonstrated the responsiveness of canine endometrial epithelial and stromal cells to oestrogen and progesterone in a monolayer cell culture system [5]. However a 3D co-culture system can much better mimic conditions present in the endometrium, due to the maintenance of epithelial cell differentiation, cell migration, cell signaling and drug responses [6-10]. The 3D co-culture system is designed to provide an appropriate microenvironment for the correct structure and function of epithelial cells, including cell-cell interactions, media, and composition of extracellular matrix (ECM), which defines cellular and tissue stiffness [10]. The structure and function of cells are closely intertwined, and therefore we used primary isolated uterine glands with their natural tissue structure featuring polarized glandular epithelial cells (GECs), surrounded by their original basement membrane, and stromal cells (SCs). The different cell types, in particular endometrial GECs, surface epithelial cells, and SCs, show strong interactions with diverse expression patterns of ERs and PRs during the canine oestrous cycle and among the different regions of the canine endometrium [11,12]. It is well known that the different cell types of the canine endometrium show different ER and PR expression patterns during the oestrous cycle in relation to fluctuations of plasma steroid concentrations [11-13]. Increased plasma oestrogen concentrations in general lead to an increased expression of ERs and PRs, whereas a rise in plasma progesterone levels is accompanied by decreased expression of ERs and PRs [11,12]. Increasing plasma oestrogen levels have been reported to lead to an increased ER expression in endometrial luminal epithelial and myometrial cells, but to a decreased ER expression in SCs and GECs [5,11,12]. It has been shown that proliferation patterns of the canine endometrium are influenced by plasma steroid hormone levels as well [14,15]. Oestrogens stimulate growth, vascularity and edema of the endometrium as well as proliferation of the glandular epithelia, whereas progesterone promotes proliferation of SCs and secretory activity of the endometrial glands [3,11,12,16]. These results underline the distinct responsiveness of the different endometrial cell populations to the respective steroid hormones. The advantages of 3D co-culture were studied in human systems with a main focus on mammary glandular epithelial cells to mimic and study the human breast in culture [17-20], as well as endometrial and ovarian cells [21,22], mainly for cancer research. In veterinary medicine only a few 3D cell cultures have been established for experimental approaches [23-26], and a cell culture system of complete endometrial glands with their specific environment has not existed until now.

The aim of our study was to apply our established 3D co-culture system, which mimics the *in vivo* canine endometrium with intact primary uterine glands in their original structural environment (basement membrane, ECM, SCs), to study the influence of steroid hormones on the uterine glands and the surrounding SCs. We hypothesized that different physiological concentrations of progesterone or oestrogens influence the expression patterns of steroid hormone receptors in these cells *in vitro.* Furthermore, the effects of these hormones on proliferative activity of the *in vitro* endometrial model were evaluated. Besides a morphological evaluation (histology and transmission electron microscopy) several markers (immunohistochemistry for β-catenin, laminin, cytokeratin, vimentin, Ki67, ER, PR) were used to verify differentiation as demonstrated by cell-cell-contacts, cytoskeleton, polarity of the cultured glandular epithelial cells, and lectin binding patterns, also in comparison with the *in vivo* situation in the canine endometrium. This 3D cell culture system allows the study of physiological and pathological mechanisms acting in the canine endometrium at the cellular level, which is almost impossible in the living animal. On the basis of the demonstrated responsiveness of the 3D cultured endometrial GECs and SCs to supplemented steroid hormones we expect this system to make a significant contribution to the knowledge about the endocrine regulation of endometrial cell populations. In addition, the development of similar 3D cultures will be applicable for the experimental investigation of other biological systems.

## Methods

### Animals and tissue sampling

Uterine tissue for the present study was collected from routine ovariohysterectomy of ten bitches of different breeds (Deer Pinscher, Beagle, Collie, Chihuahua, Yorkshire Terrier, Pekinese, Great Dane and two mongrel) and ages (mean age: two years, range: 1–5 years). Surgery was performed under general anesthesia at the Department of Companion Animals and Horses, Section of Obstetrics, Gynecology and Andrology of the University of Veterinary Medicine at Vienna, Austria and at a veterinary practice in Vienna, Austria.

Tissue sampling and evaluation as well as anonymized publication of the received data were in accordance with the pet owners and the project was approved by the local ethical commission at the Vetmeduni Vienna (ETK) to be based on the respective regulations of good scientific practice.

The dissected uterine tracts were transported in sterile Dulbecco’s Phosphate Buffered Saline (DPBS-AB, without Ca & Mg; PAA Laboratories, Pasching, Austria) containing 0.5% Gentamicin (PAA Laboratories) and 1.5% Nystatin suspension (10,000 units/mL in DPBS; Sigma Aldrich, Vienna, Austria), at 4-8°C.

### Native tissue histology

Visceral fat was removed from the uterine tracts under sterile conditions. For the comparison between *in vivo* endometrial tissue and cultured uterine glands, samples (1 cm^3^) of the uterine horns (cranial and caudal regions) as well as of the uterine body (bifurcation) were separated and immersion fixed in 4% buffered formaldehyde for 24 to 48 hr at 4°C and then embedded in Paraplast^®^ (Vogel, Giessen, Germany)*.* Samples from the same regions were reduced to 1–2 mm^3^ and separated for transmission electron microscopy.

For the determination of the oestrous cycle stage, histological sections of 2 μm thickness were cut and stained with haematoxylin and eosin (H&E) according to Romeis [27]. Histological evaluation of the stage of the oestrous cycle was performed according to Barrau *et al.*[16] and Galabova *et al.*[14]. Furthermore, immunohistochemical oestrogen and progesterone receptor expression patterns of the endometrial tissue were evaluated for cycle stage determination according to Vermeirsch *et al.*[12,28], and allocation of proliferative activity was assessed by anti-Ki67 staining, according to van Cruchten *et al.*[15].

### 3D cell culture

#### Isolation of uterine glands and stromal cells

Uterine tissue preparation for 3D cell culture was performed according to Stadler *et al.*[4] with modifications. In brief, the uterine tissue was rinsed with DPBS-AB and cut into pieces of about 2 cm^2^ with sterile scalpel blades. These pieces were opened longitudinally and then cut into further pieces (2–3 mm^2^). Subsequently, the minced tissue was placed into a Petri dish (80 cm diameter) and gently rinsed three times with DPBS-AB to remove the majority of erythrocytes. Subsequently, pieces were placed into a glass beaker containing standard medium [88% medium M199 with L-glutamine, 10% fetal calf serum FCS, 1% antibiotic-antimycotic solution (PAA Laboratories, Pasching, Austria) and 1% Fungizone^®^, liquid (GIBCO / Life Technologies, Austria)] with 1 mg collagenase I / mL (from *Clostridium histolyticum*, prepared from Type XI, Sigma-Aldrich, Vienna, Austria) and a sterile magnetic stirrer for tissue disintegration. The mixture was mildly stirred for 4–6 hr (depending on the tissue structure of the obtained uterus) in a tissue culture incubator at 37°C and 5% CO_2_. Subsequently, the solution was filtered through a stainless steel filter (pore size 280 μm; Bellco Glass, Dunn Labortechnik, Asbach, Germany) to remove tissue parts that had not disintegrated. The filtered solution containing uterine glands and stromal cells was further filtered through a cell filter (pore size 40 μm, BD Falcon™, Becton Dickinson GmbH, Heidelberg, Germany) into a sterile Petri dish. Isolated stromal cells passed through the filter whereas glandular structures remained on the filter mesh and were washed into a separate Petri dish with standard medium. Stromal and glandular fractions were centrifuged in a sterile 15 mL tube for one minute at 0.6 × g. Each pellet was resuspended in 1 mL sterile distilled water for 30 s to eliminate remaining erythrocytes. Afterwards, pellets were washed in 10 mL standard medium and the fractions were centrifuged again. The supernatant was removed and the pellets were resuspended in hormone-free medium (stromal fraction with 1300 μl, glandular fractions with 5200 μl).

The hormone-free medium was prepared by filtration of standard medium through a Sep-Pak C18 column (Sep-Pak C18 Classic Type, Waters, Vienna, Austria) prepared as specified by the manufacturer, to extract present steroids from the standard cell culture medium containing fetal calf serum, to enable the application of specified hormone concentrations in the cell culture system. The concentrations of E and P in the standard medium (M199 + 10%FCS) were among 11 pg/mL E and 25 pg/mL P, determined with a competitive enzyme immunoassays using biotin linked steroids [29]. After filtration through the Sep-Pak C18 column neither estrogens nor 20-oxo-gestagens (including progesterone) were present in the hormone free medium.

At this preparation stage, glandular (200 μl) and stromal (50 μl) fractions were collected for histological and electron microscopical evaluation of the morphological state of the cells after the isolation procedure.

#### Experimental design

Experiments were performed on Matrigel™ 24-well plates (BD BioCoat 24-well Multiwell Plates, Becton Dickinson GmbH, Heidelberg, Germany), thawed from -20°C to 4°C for 5–7 hr*.* After the thawing process, six wells were rehydrated with 1 mL standard medium, 18 wells with 1 mL hormone free medium for 30 min at 37°C. Media were removed and 750 μl fresh medium (6× standard medium, 18× hormone-free medium) were added to each well, and plates were ready to use. Plates were provided with 200 μl glandular fraction and 50 μl stromal fractions in each well.

Three different physiological concentrations of the steroid hormones 17β-estradiol (Sigma Aldrich, Germany; E; 15, 30, and 100 pg/mL, i.e. 5.5×10^-2^, 11×10^-2^, and 36.7×10^-2^ nM, respectively) and progesterone (Sigma Aldrich, Germany; P; 3, 15, and 30 ng/mL, i.e. 9.54, 47.7, and 95.4 nM respectively) were tested. On each 24-well plate, 6 wells prepared with hormone-free medium were supplemented with the respective hormone solution (10 μL/mL medium) of one concentration. Six wells with standard medium and 6 wells with hormone-free medium served as controls. 3D co-cultures with uterine glands and stromal cells were incubated for either 24 hr or 48 hr in each medium group. Medium was changed after 24 hr, including two washing steps with pre-warmed DPBS. Each hormone concentration was tested in three independent experiments (tissue originating from three different dogs).

Additional experiments (12 wells) with standard medium containing 10 μl sterile distilled water / mL represented the control for the solvent of the steroid hormones.

#### Preparation of 3D co-culture samples

To harvest the cultured uterine glands and stromal cells, the Matrigel™ gel was gently mixed with the medium in the well and then aspirated using a plastic pipette (tip diameter 2–3 mm) to ensure the integrity of the glandular structures. The gel containing the glandular structures and stromal cells was transferred to a 2 ml tube, centrifuged (3 min, 0.84 × g) and the gelatinous pellet was treated with the respective fixative. At each time point, one well was used for electron microscopy and two wells were pooled for histological preparation.

#### Histology

For histological preparation samples were fixed for 24 hr at 4°C, then centrifuged again (3 min, 0.84 × g) and pellets were embedded in Histogel^®^ (Richard-Allan Scientific, Microm International, Walldorf, Germany; as specified by the manufacturer) and subsequently in Paraplast^®^ (Vogel) by means of an automatic embedding device. Serial sections of 3 μm thickness were cut and stained with H&E according to Romeis [27].

#### Immunohistochemistry

Serial sections (3 μm) of native tissue and 3D cultures were mounted on APES/glutaraldehyde -coated slides. Endogenous peroxidase activity was blocked by incubation in 0.6% H_2_O_2_ in methanol for 15 min at room temperature. A protein block (1.5% normal goat serum) was used to minimize unspecific binding of the primary antibody. The unlabeled primary antibodies (anti-oestrogen receptor, anti-progesterone receptor, anti-Ki-67; for sources, pre-treatments and dilutions see Table 1) were detected with the ImmunoVision secondary system (ImmunoVision Technologies, Brisbane, CA, USA) using DAB (3,3’diaminobenzidine-tetrahydrochloride substrate in Tris buffer pH 7.4 and 0.03% H_2_O_2_) as chromogen. Finally, slides were washed with distilled water, counterstained with haemalum, dehydrated and mounted by use of xylene-soluble medium (DPX, Fluka, Buchs, Switzerland). For the fluorescent detection (anti-cytokeratin, anti-vimentin, anti-laminin, anti-β-catenin) Alexa Fluor™ 488 goat anti-mouse (Molecular Probes, Eugene, OR, USA; dilution 1:100) secondary antibody was used and nuclear counterstaining was performed with 4’,6-diamidino-2-phenylindole (DAPI, Molecular Probes / Life Technologies, Vienna, Austria).

**Table 1:** Sources, pre-treatments and dilutions of the antibodies used in this study

**Antibody**	**Source**	**Clone**	**Pre-treatment**	**Dilution**
Anti-oestrogen receptor	Zymed / Life Technologies, USA	Poly rabbit	Nuclear Decloaker. Biocare Medical. Concord. CA. USA. pH 9.5. 3× 5 min.	1:200
Anti-progesterone receptor	Immunotech SAS, Marseille, France	10A9 rabbit	Boil in citrate buffer, pH 6.0, 4× 5 min.	1:200
Anti-Ki-67	Thermo Fisher Scientific, Fremont, CA, USA	SP6 rabbit	Boil in citrate buffer, pH 6.0, 3× 5 min.	1:400
Anti-cytokeratin	Sigma-Aldrich, Germany	8.13 mouse	Boil in citrate buffer, pH 6.0, 2× 5 min.	1:100
Anti-vimentin	Dako, Glostrup, Denmark	V9 mouse	No pre-treatment	1:400
Anti-laminin	Dako, Glostrup, Denmark	Poly rabbit	Protease (Sigma) 1mg/mL PBS, 20 min, room temp.	1:500
Anti-β-catenin	Acris Antibodies, Herford, Germany	9G2 mouse	Boil in citrate buffer, pH 6.0, 3× 5 min.	1:100

Negative controls were performed by substitution of the primary antibodies with PBS. Evaluation of the sections was performed using light microscopy (Polyvar, Reichert-Jung, Vienna, Austria) and confocal laser scanning microscopy (Zeiss, LSM 510 Meta, Vienna, Austria).

#### Lectin histochemistry

Paraffin sections were pre-treated as described by Bartel *et al*. [30] and incubated with the respective biotinylated lectin (Ulex Europaeus Agglutinin – UEA I, Wheat Germ Agglutinin -WGA; Helix Pomatia Agglutinin - HPA, Vector Laboratories, Burlingame, CA, USA) at a concentration of 10 μg/mL. After incubation, sections were washed in PBS solution and incubated with avidin-biotin-peroxidase complex (Vectastain ABC Kit, Vector Laboratories) according to the manufacturer’s instructions, then washed and developed with DAB.

In case of double labelling, fluorescent Alexa Fluor™ 488 goat anti-mouse secondary antibody was used for antigen (β-catenin) detection, and streptavidin (Molecular Probes, Eugene, OR, USA, 568 nm, red) for lectin (UEAI, HPA, WGA) demonstration.

#### Transmission electron microscopy

Samples of native tissue and 3D cultures were fixed in 3% buffered glutaraldehyde (pH 7.4) at 4°C for at least 12 hr, flushed three times with phosphate buffer, and post-fixed in 1% OsO_4_ for 2 hr at room temperature, followed by washing with phosphate buffer. Dehydration was performed in a series of graded ethanol solutions. Infiltration with propylene oxide was followed by increasing ratios of epoxy resin: propylene oxide (1:1, 3:1) and finally pure resin. After two additional changes, the resin was polymerized at 60°C. Semithin sections (0.7 μm) were stained with toluidine blue and embedded with DPX (Fluka, Buchs, Switzerland). Ultrathin sections were cut at 70 nm, contrasted with alkaline-lead citrate and methanolic-uranyl acetate and viewed with a Zeiss EM 902 electron microscope and a SiS-software assisted CCD camera (Nikon).

#### Scoring and statistical analysis

The scoring of proliferative activity (anti-Ki-67 staining) as well as ER and PR expression was performed on two slides for each sample, counting in three different randomly-selected areas (40× magnification) using a light microscope with a Nikon DS-Fi1 digital camera system and Nikon NIS-Elements imaging and counting software (Nikon). The number of nuclei positive for the respective immunohistochemical staining was correlated to the total number of cells counted in that area. Glandular epithelial cells and stromal cells were counted separately. A minimum of 50 cells for cell culture samples, and 100 cells for native tissue samples were used per area.

The statistical analyses were performed using IBM^®^ SPSS^®^ Statistics 20 software. The assumption of normal distribution was tested using the Kolmogorov-Smirnov test. All data were normally distributed. Values are expressed as means ± standard deviations (SD). To analyze the effects of time, medium and concentration on the expression of ER and PR as well as proliferative activity, a linear mixed model was performed appointing time and media as repeated measures. The significance of the differences between factor levels was determined using Bonferroni alpha correction as a post hoc procedure. For all analyses, a p-value < 0.05 was considered significant.

## Results

### Histological and immunohistochemical evaluation of the native endometrial tissue

**Figure 1:**
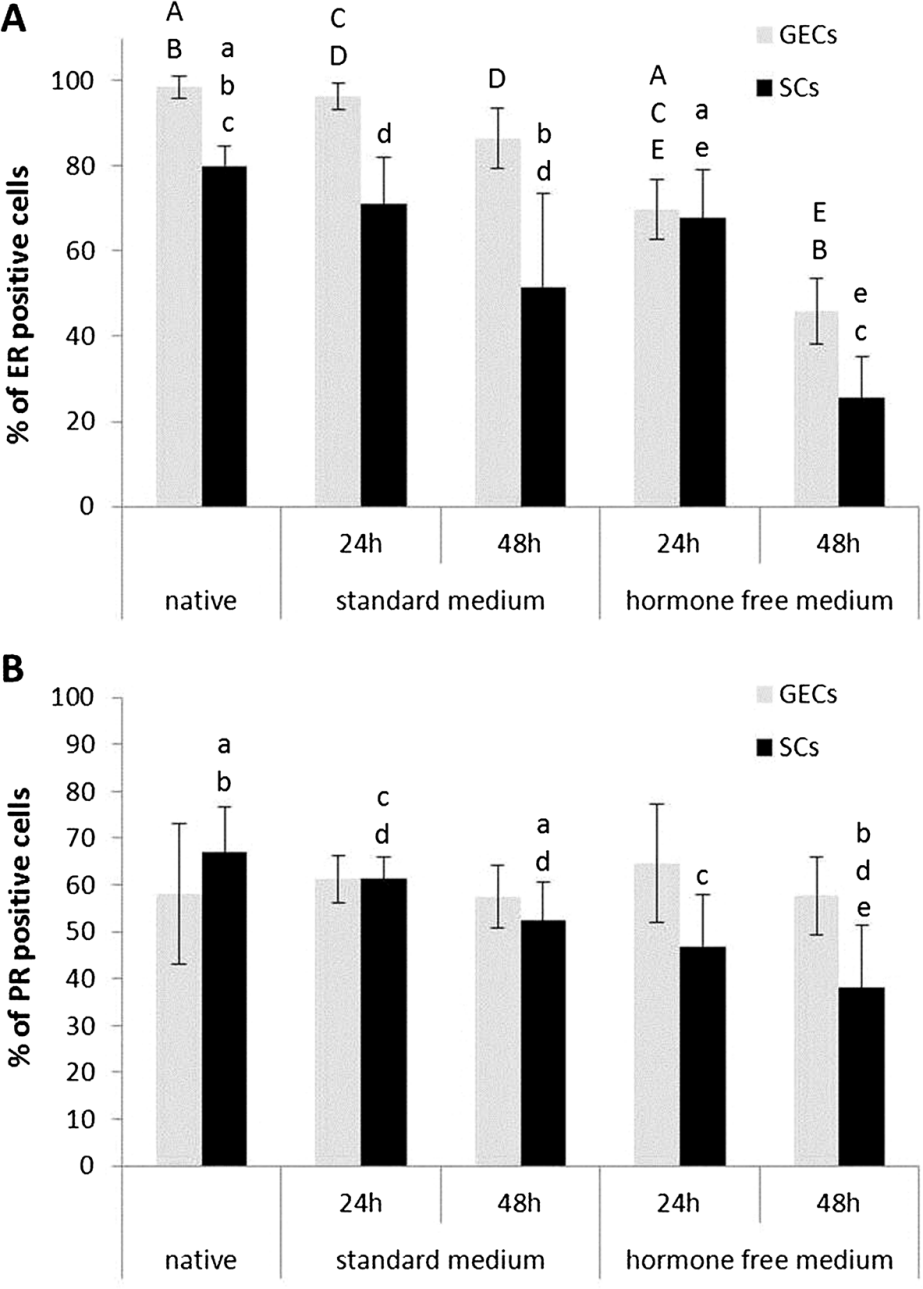
**Estrogen and progesterone receptor expression in 3D cultured and native canine endometrial cells. **Percentage of oestrogen receptor ER (**A**) and progesterone receptor PR (**B**) positive glandular epithelial cells (GECs) and stromal cells (SCs) in the canine uterine tissue of origin (native) as well as after 24 and 48 hr of cell culture in standard medium and hormone free medium. Statistical significances (p < 0.05) are indicated as letters (**A**-**E **for GECs, a-e for SCs) above the related columns.

**Figure 2:**
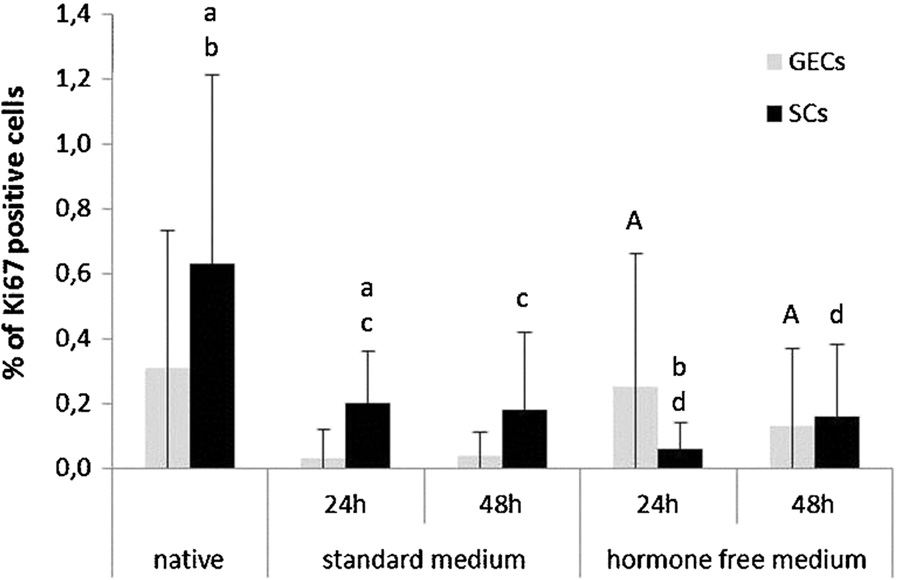
**Proliferative activity in 3D cultured and native canine endometrial cells. ** Percentage of anti-Ki67 positive glandular epithelial cells (GECs) and stromal cells (SCs) indicating proliferative activity in the canine uterine tissue of origin (native) as well as after 24 and 48 hr of cell culture in standard medium and hormone free medium. Statistical significances (p < 0.05) are indicated as letters (A for GECs, a-d for SCs) above the related columns.

The canine uterine tissues used in this study for the 3D cell culture systems were classified as late metestrous (n = 3) and anestrous (n = 6) by means of histological and immunohistochemical assessment, including oestrogen and progesterone receptor expression (Figure 1) and proliferative activity (Figure 2).

### Histological and immunohistochemical evaluation of the 3D cultured endometrial glands and stromal cells

**Figure 3:**
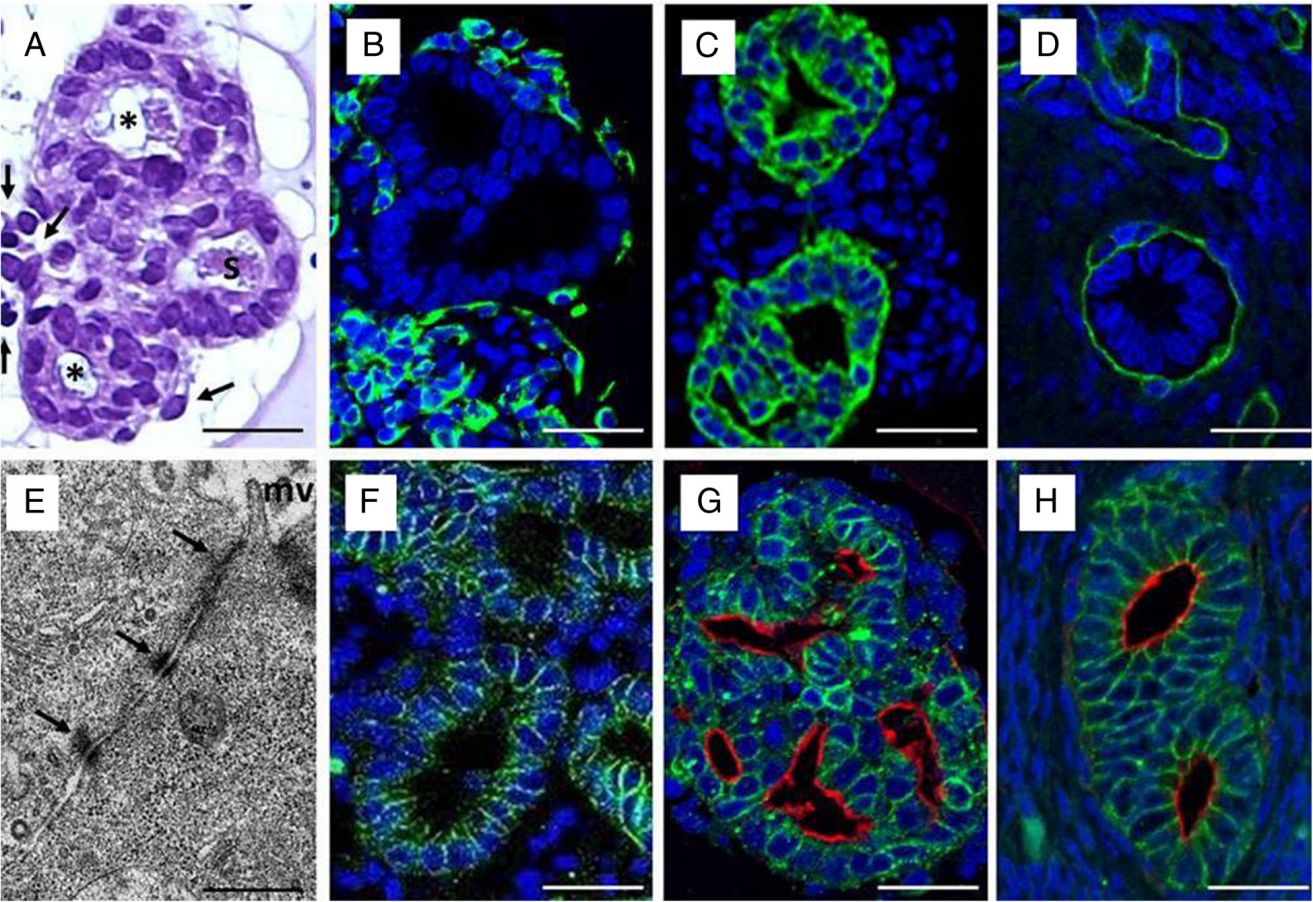
**A-H: 3D cell culture of canine endometrial glands with surrounding stromal cells cultured for 48 hr in standard medium (M199 + 10% FCS). **(**A**) H&E-stained histological section of 3D-cultured canine endometrial glands (asterisk) and surrounding stromal cells (arrows) featuring physiological morphology with luminal formation and secretory activity(s). (**B**) Immunohistochemical characterization of stromal cells by anti-vimentin staining (green); glandular epithelial cells lack any staining reaction,counterstained with DAPI (blue). (**C**) Glandular epithelial cells were identified by immunohistochemical anti-cytokeratin staining (green), counterstained with DAPI (blue). (**D**) Demonstration of the intact basement membrane (green) surrounding the glandular structures with anti-laminin immunohistochemical staining, counterstained with DAPI (blue). (**E**) Transmission electron micrograph of 3D-cultured canine endometrial epithelial cells to demonstrate apical polarity (mv, microvilli)and intact junctional complex (arrows) featuring tight junctions, zonulaadherens and desmosomes, characteristic for differentiated glandular epithelial cells. (**F**) Basolateral polarization of the 3D-cultured glandular epithelial cells, ensuring correct luminal formation and function,demonstrated with anti-b-catenin immunohistochemical staining (green),counterstained with DAPI (blue nuclei). (**G**) Combined demonstration of apical polarization, with histochemical WGA lectin binding to the glycocalyx (red), and basolateral polarisation demonstrated with immunohistochemical anti-b-catenin staining (green) on 3D-cultured canine endometrial glands(nuclei demonstrated by DAPI counterstaining, blue). (**H**)The same combined double-staining method (compare with panel G)of the canine endometrium in vivo, featuring apical and basolateral polarization of the endometrial glands demonstrated histochemically byapical WGA lectin binding to the glycocalyx (red), and immunohistochemically bybasolateralanti-b-catenin staining (green),comparable to the 3D in vitro cultured glandular structures (panel G); DAPI counterstaining (blue). Scale bars A-D, F-H25 µm; E 1 µm.

H&E-stained histological sections of the 3D-cultured glandular structures surrounded by stromal cells did not show any significant structural changes during the culture period of 48 hr. Luminal secretions appeared in all glandular structures indicating grossly correct physiological function of the glandular epithelial cells (Figure 3A).

To distinguish the phenotypes of the different isolated primary endometrial cell populations (glandular epithelial cells and stromal cells) as well as to examine the structural and functional characteristics of the cells, the expression of a series of key markers was assessed by means of immunohistochemistry.

Vimentin was used as a mesenchymal marker to identify stromal cells in the native tissue and the cultured endometrial structures, and to distinguish them from epithelial cells, which were used as an internal negative control (Figure 3B). Cytokeratin, used to characterize glandular epithelial cells, identified clear lumen formation and the cytoskeletal composition of the GECs without staining the stromal cells (Figure 3C). Laminin was used to assess the presence of the basement membrane surrounding the endometrial glands that is important for the tissue-specific structure and function correlated to the basolateral and apical polarization of the glandular epithelial cells (Figure 3D). Apical polarization of the 3D-cultured glandular epithelial cells was demonstrated by transmission electron microscopy (TEM), showing microvilli at the apical luminal region of the glandular epithelial cells and cell-cell contacts characteristic for glandular epithelial cells (tight junctions, zonula adhaerens, desmosomes) (Figure 3E).

Immunohistochemical anti β-catenin staining demonstrated the basolateral domains of the cell membranes of GECs, and was applied to detect β-catenin as a protein bound to the cytoplasmic domain of E-cadherin (Figure 3F). This protein complex is important for cell-to-cell adhesions, and this correlates with correct lumen formation of the GECs *in vivo* and *in vitro*. To demonstrate functional apical polarity of the glandular epithelial cells, different lectins (UEA I, HPA and WGA) were applied to analyse surface glycoconjugates. In contrast to that of the other lectins, WGA-binding, with strong affinity to N-acetylglucosamine sugar residues of the glycocalyx, remained stable during the whole culture period in the standard and hormone-free medium, and was therefore chosen to test the apical polarization of the glandular epithelial cells. Based on these results, a new double-staining method using β-catenin immunohistochemistry in combination with WGA lectin histochemistry was established in our laboratory to demonstrate apical and basolateral polarization of the 3D-cultured canine endometrial glandular structures (Figure 3G); this method showed the 3D-cultured structures to be comparable with those found in the native tissue (Figure 3H).

The above-mentioned immunohistochemical markers were used as quality assurance for the cell-specific characteristics. The protein expression patterns of these markers did not show any alterations, either in the standard medium, or in any treatment group, compared to the original endometrial tissue during the whole culture period.

**Figure 4:**
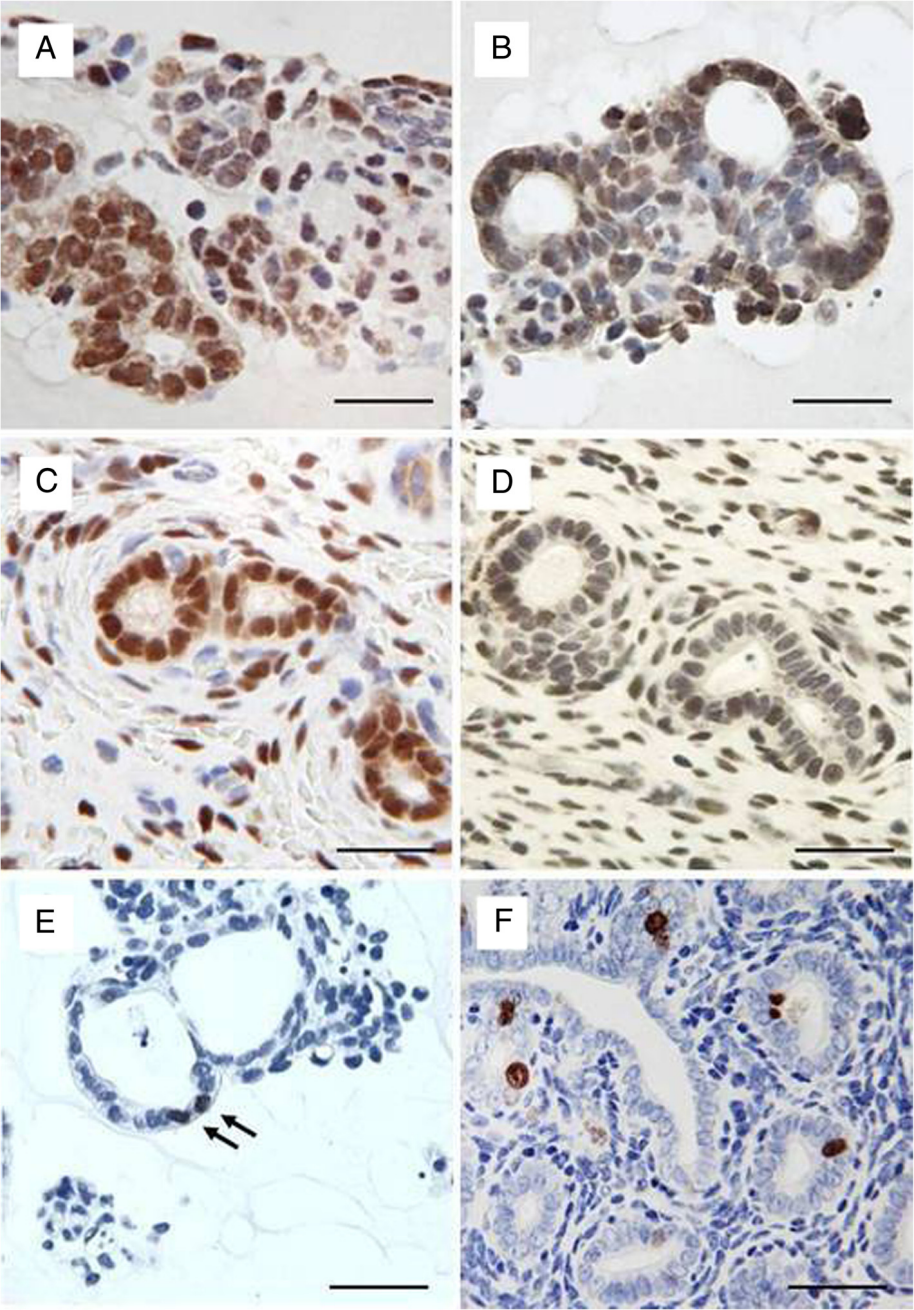
**Immunohistochemical demonstration of oestrogen and progesterone receptorexpression and proliferative activityin 3D cell cultured canine endometrial cells compared to the native tissue. **Oestrogen (A) and progesterone (B) receptor expression of glandular epithelial and stromal cells cultured for 48 hr in standard mediumcompared to the native tissue (Coestrogen receptor, Dprogesterone receptor) indicated by immunohistochemical nuclear staining. Proliferative activity in 3D cell-cultured canine glandular epithelial cells (E) (arrows) compared to the in vivo situation (F) of uterine glands and surrounding stromal cells by means of anti-Ki67 immunohistochemical staining. Scale bars A-F, 25µm.

Staining patterns of nuclear oestrogen- and progesterone-receptors in the canine endometrial glandular epithelial and stromal cells cultured in standard medium (Figure 4A and 4B) were comparable to those in native uterine tissue samples (Figure 4C and 4D). Low proliferative activity, as demonstrated by immunohistochemical anti-Ki-67 staining, was detected in both 3D-cultured endometrial glands and stromal cells (Figure 4E) and glandular epithelium and stromal cells in native uterine tissue samples (Figure 4F).

### Scoring and statistical analysis of steroid hormone receptor expression and proliferative activity of endometrial glands and stromal cells in response to hormone supplementation in the 3D culture

Glandular structures and surrounding stromal cells cultured in standard medium for 24 hr maintained their characteristic expression patterns concerning ER and PR comparable to the *in vivo* situation of the tissue of origin (Figure 1). After 48-hr culture in the standard medium, ERs of both cell types decreased, whereas PR expression was mainly reduced in SCs (Figure 1). Proliferative activity was below 1% in both standard medium cultured cells and in the native uterine tissue (Figure 2).

To exclude potential effects of the supplemented FCS, with its undefined components, the standard medium was filtered through a Sep-Pak C18 column to remove steroid-based molecules (hormone-free medium). ER expression patterns in GECs and SCs declined after 48 hr culture in hormone-free conditions, whereas PRs declined only in SCs significantly (Figure 1). Proliferative activity was below 0.4 % in both cell types after a 48-hr hormone-free culture period (Figure 2). These hormone-free medium conditions were then used as baselines (control group) for testing the effects of steroid hormones on ER and PR expression, and on proliferative activity.

### Effects of different concentrations of 17β-estradiol on ER and PR expression and proliferative activity of endometrial glands and stromal cells in 3D culture

**Figure 5:**
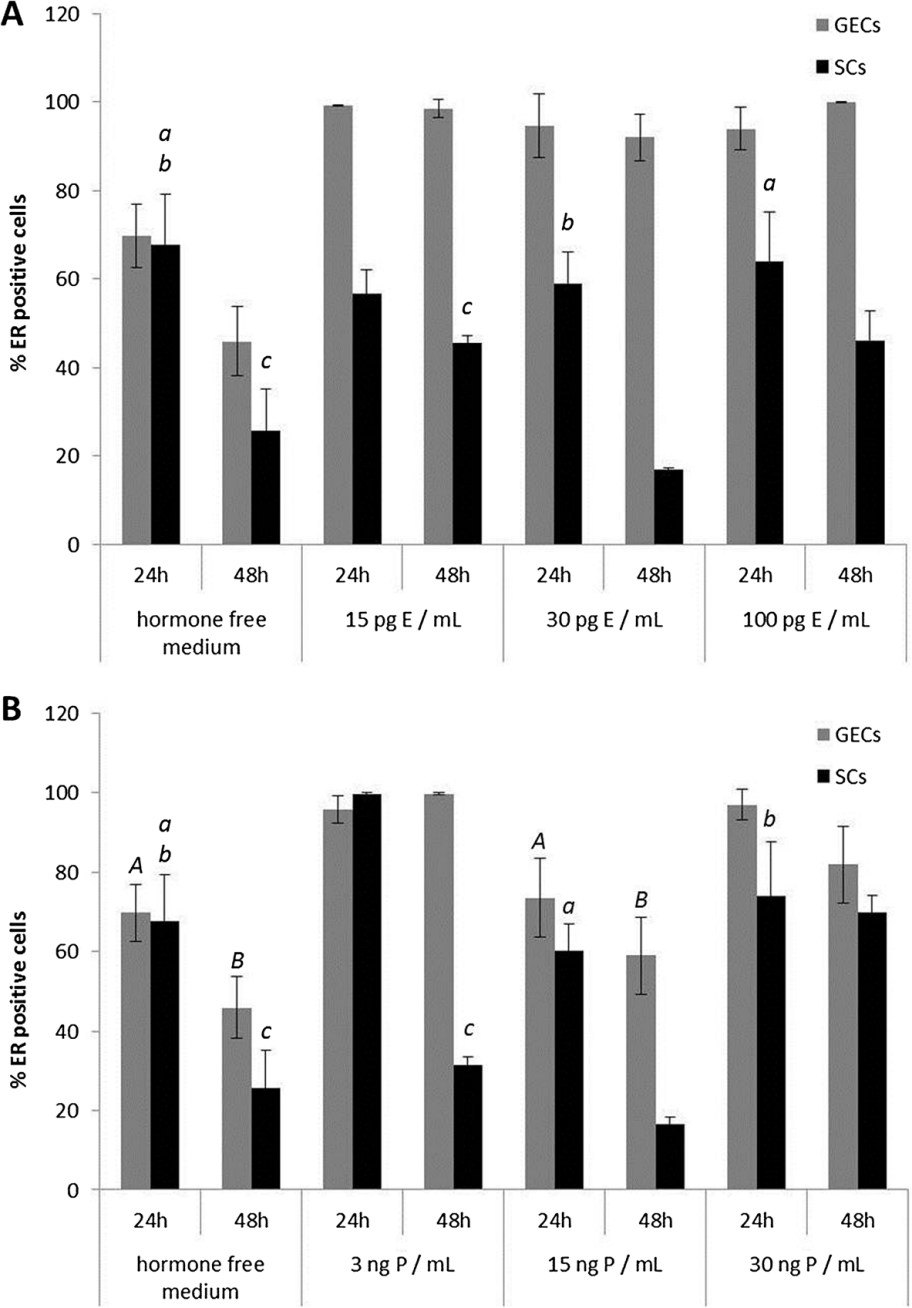
**Alterations of oestrogen receptor expression in 3D cultured canine endometrial cells due to 17β-estradiol and progesterone supplementation. **Percentage of oestrogen receptor ER positive glandular epithelial cells (GECs) and stromal cells (SCs) after 24 and 48 hr of 3D cell culture with (A) 17β-estradiol supplementation (E) and (B) progesterone (P) in the respective three different concentrations. Due to the high number of statistical significant results concerning the comparison with the control group (hormone free) statistical *non-significant *(p > 0.05) results are indicated as *italic *letters (*A-B *for GECs, *a-e* for SCs) above the related columns.

**Figure 6:**
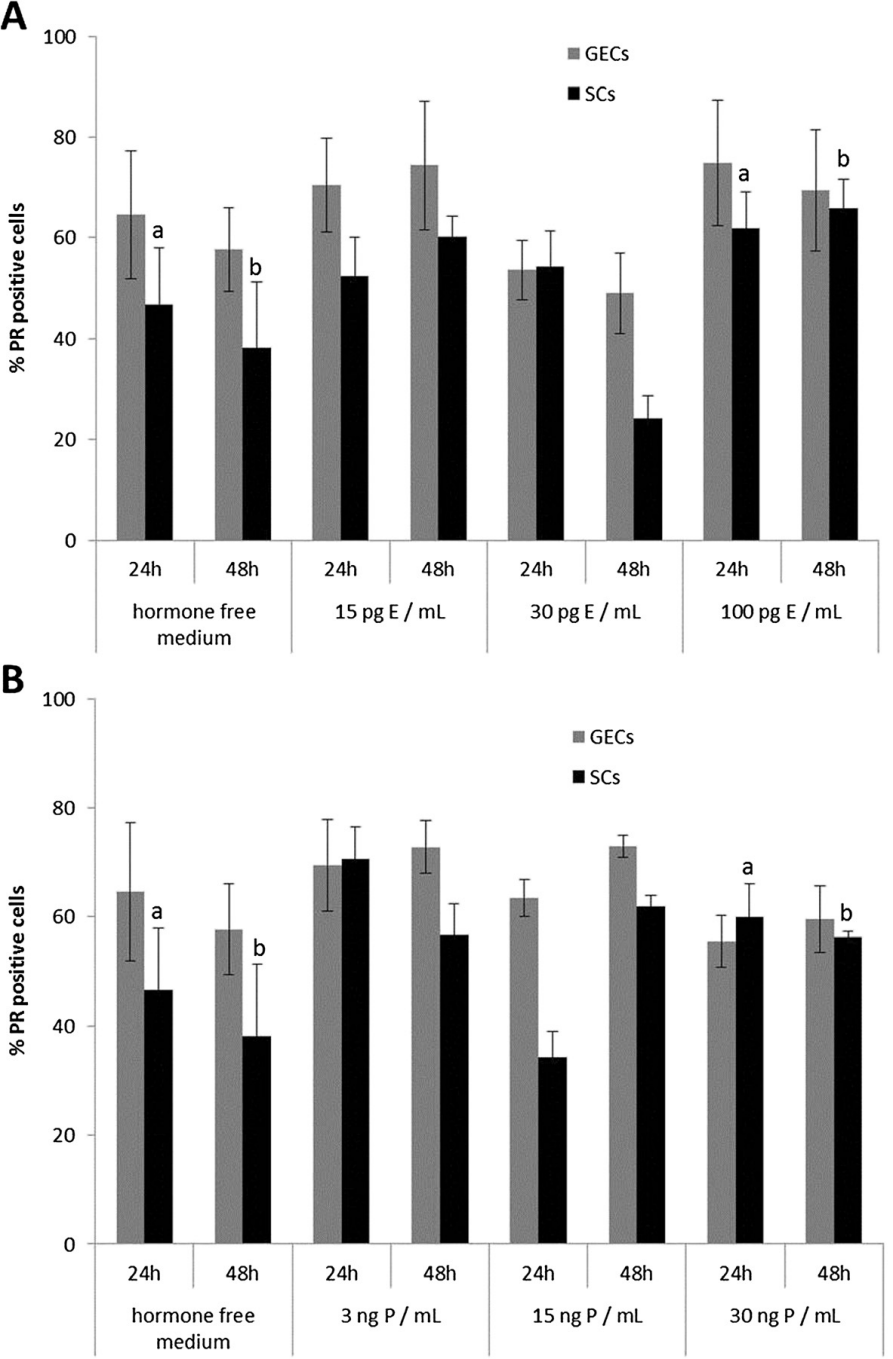
**Alterations of progesterone receptor expression in 3D cultured canine endometrial cells due to 17β-estradiol and progesterone supplementation. **Percentage of progesterone receptor PR positive glandular epithelial cells (GECs) and stromal cells (SCs) after 24 and 48 hr of 3D cell culture with (A) 17β-estradiol (E) and (B) progesterone (P) supplementation in the respective three different concentrations. Statistical significances (p < 0.05) are indicated as letters (A-E for GECs, a-e for SCs) above the related columns for comparison with the control group (hormone free medium).

Different concentrations of 17β-estradiol (E) induced significant changes in the expression patterns of ER and PR in the 3D-cultured endometrial glandular epithelial cells and stromal cells during the 24- and 48-hr culture period compared to the control group (hormone free medium) (Figures 5A and 6A). E supplementation had a significant stimulatory effect on ERs in GECs in all concentrations. In SCs ER expression was reduced after treatment with 30 pg/mL E for 48 hr. PR expression in GECs increased after 24-hr culture time with 15 pg/mL and 100 pg/mL E but decreased due to supplementation of 30 pg/mL E (Figure 6A). In SCs an increase of PRs was observed after 24 hr in all of the treatment groups but after supplementation of 30 pg/mL E for 48 hr PRs of the SCs declined compared to the control group (Figure 6A).

Beside the comparison with the control group significant results were also obtained between the different concentration groups of E supplementation. The effects of the different concentrations of E on ER expression were mainly present in SCs with a significant reduction between 15 and 30 pg/mL E (p < 0.01) and a significant increase of ER positive SCs between 30 to 100 pg/mL E (p < 0.01) after 48-hr culturing period. Furthermore, PR expression in SCs was reduced after 48hr - treatment with 30 pg/mL E compared to the expression levels in SCs treated with 15 (p < 0.01) and 100 pg/mL E (p < 0.01), respectively, for the same culturing period.

**Figure 7:**
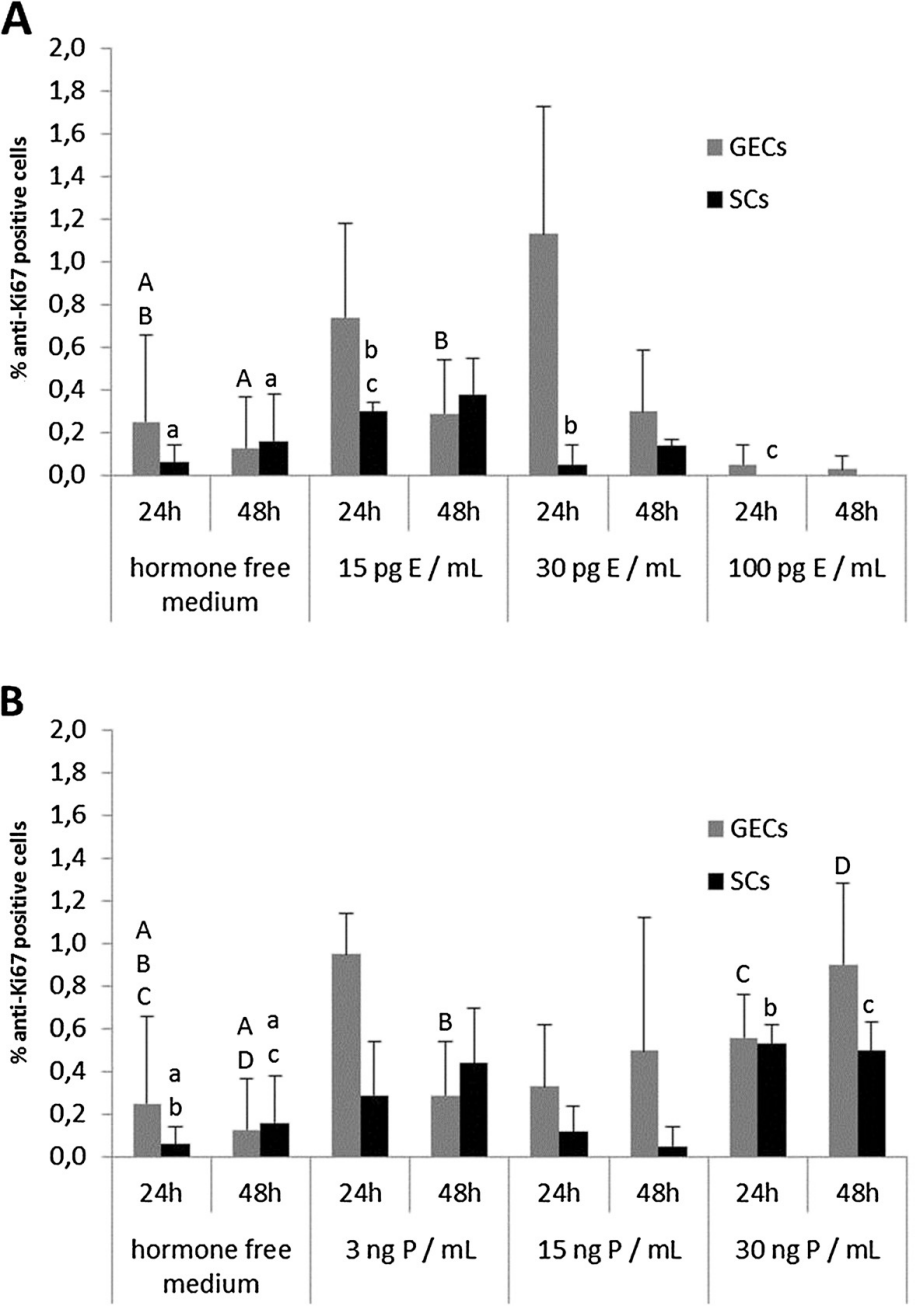
**Alterations of proliferative activity in 3D cultured canine endometrial cells due to 17β-estradiol and progesterone supplementation. **Percentage of anti-Ki67 positive glandular epithelial cells (GECs) and stromal cells (SCs) indicating proliferative activity after 24 and 48 hr of 3D cell culture with (A) 17β-estradiol (E) and (B) progesterone (P) supplementation in the respective three different concentrations. Statistical significances (p < 0.05) are indicated as letters (A-E for GECs, a-e for SCs) above the related columns.

The proliferative activities of GECs and SCs were assessed using an anti-Ki67 based assay. Although for both cell types an increase in Ki67-positive cells was measured following treatment with 30 pg/mL and 15 pg/mL E for 24 hr compared to hormone-free cultured cells, a significant decrease in SCs was demonstrated with 100 pg/mL E (Figure 7A).

### Effects of different concentrations of progesterone on ER and PR expression and proliferative activity of endometrial glands and stromal cells in 3D culture

The application of progesterone to 3D-cultured GECs and SCs induced significant changes in the expression patterns of the ER and PR during a 24-hr and 48-hr culture period compared to the control group, closely depending on the concentration of the supplemented P (Figures 5B and 6B).

In all concentrations P had a stimulatory effect on ER expression in GECs compared to the control group (Figure 5B). In SCs an increase of PRs was observed after 3 ng/mL and 30 ng/mL P treatment but due to supplementation of 15 ng/mL P PRs in SCs decreased. P had a reducing effect on PR expression in GECs after 24-hr culturing period compared to the control group, but after 48-hr culturing period PRs increased in GECs. In the SCs a reducing effect of only 15 ng/mL P after 24 hr treatment was observed followed by an increase of PRs in all treatment groups compared to the control group after 48 hr culturing time (Figure 6B).

The clear dose dependent effects of P on ER expression in GECs were demonstrated by the reduction of ER positive GECs after 15 ng/mL P treatment compared to the increased amounts of ER positive GECs after 3 ng/mL P (p = 0.016) and 30 ng/mL P (p = 0.013), respectively, 24 hr-treatment. In the SCs ER expression was reduced due to supplementation of 15 (p < 0.01) and 30 ng/mL P (p = 0.035), respectively, compared to the high expression levels in SCs with 3 ng/mL P treatment after 24 hr-culturing period. After 48hr-culturing period with 15 ng/mL P ERs in GECs were reduced compared to the 3 (p < 0.01) and 30 ng/mL P (p = 0.036) treatment. In SCs comparable results were obtained with a significant reduction of ERs after 48hr - culturing period with 15ng/mL P compared to 3 (p < 0.01) and 30 ng/mL P (p < 0.01), respectively. The high amount of ER positive SCs after 48hr - treatment with 30 ng/mL compared to 3 ng/mL P (p < 0.01) reflects the dose- and time-dependent reactions of SCs to P.

For all concentrations of P, proliferative activity of GECs and SCs was below 1%, but showed indication of a small enhancement, following treatment with 3 ng/mL and 30 ng/mL P for 24 hr, relative to the hormone-free medium controls (Figure 7B). SCs treated with 15 ng/mL P for 48 hr showed a reduction in PR expression compared to the control group.

Detailed results, including all percentage values with their ± SDs, are listed in Additional file 1: Table S1.

## Discussion

### The canine organotypic endometrial model features differentiated glandular structures with proper cell characteristics

The application of a 3D organotypic endometrial culture system using differentiated glandular structures and surrounding stromal cells, simulating their natural environment, provides a system closely mimicking the *in vivo* situation of the endometrium, and is well suited for the study of hormonal effects on a cellular level. It is well known that unphysiological cell culture conditions lead to significant alterations of cell characteristics. Galabova *et al.*[5] described vimentin expression in canine endometrial epithelial cells *in vitro*, a common phenomenon known for monolayer cell cultures [31]. This switch in cytoskeletal protein expression from epithelial cytokeratin to mesenchymal vimentin is most probably induced by the lack of a basal lamina, crucial for the formation of tissue-specific form and function [32] and epithelial polarization [10,32,33]. In our 3D cell culture system neither changes in epithelial cytokeratin expression nor the disappearance of basal lamina structures were detected during the culture period, indicating that the glandular cells were fully differentiated. This underlines the preservation of the original basal lamina of the uterine glands, as ensured by our protocol, as an essential factor. Furthermore, we tested different lectins (UEA I, HPA, WGA) for their suitability as markers for the correct apical polarization of the GECs, and we found WGA to be the most suitable lectin, labelling the apical cell membrane reliably. Similarly, N-acetylglucosamine has recently been reported to be present on the endometrial surface and glandular epithelial cells during the physiological canine oestrous cycle as well as in pathological alterations of the uterine tissue [34]. Based on this knowledge and our results, we decided to use WGA as a marker for apical polarity of the glandular epithelial cells in our 3D cell culture system. Together with β-catenin we created a new double-labelling combination to assay apical and basolateral polarisation and using this were able to demonstrate the differentiation of cultured GECs in the 3D system. Loss of polarity is generally associated with increased proliferation and migration, two factors related to malignant alterations in tissues and dedifferentiation processes in monolayer or co-culture systems of primary cells [10,35].

### In the 3D endometrial cell culture system ER and PR expression patterns in endometrial glands and stromal cells follow exogenous hormonal stimulation

In the dog, several studies have been performed to elucidate the specific effects of steroid hormones on the different cell types of the endometrium *in vivo*[36-38]. Even with progesterone concentrations at physiological levels, altered steroid hormone receptor expression patterns in the uterus are suggested to be involved in the pathogenesis of serious uterine diseases, such as cystic endometrial hyperplasia and pyometra [1,3,39,40]. Therefore, knowledge about the direct effects of different progesterone and oestrogen concentrations on endometrial epithelial and stromal cells could help the understanding of physiological and pathological processes in the endometrium.

Compared to the initial situation of ER and PR status in the uterus, ER expression of GECs and SCs declined after a 48 hr hormone-free culture period, whereas PR expression was nearly stable in GECs and declined only in SCs. These effects were also observed in extenuated patterns in standard medium. We interpret this observation as a response to the lack of a stimulus under these culture conditions. Consistent with our observations, Pierro *et al.*[41] described altered ER expression patterns and responsiveness of human endometrial epithelial and stromal cells to E *in vitro* as a result of missing paracrine factors (e.g. insulin).

The application of different physiological concentrations of steroid hormones according to the canine oestrous cycle [42,43] demonstrated the capability of the endometrial GECs and SCs to respond to these supplements in an organotypic endometrial cell culture model. In our 3D cell culture system addition of E resulted in increased ER expression in GECs (100 pg/mL and 15 pg/mL E), but this was decreased in SCs with a minimum level following 30 pg/mL E for 48 hr. These results are at least in part comparable with the physiological situation. In the native canine endometrial tissue ER expression in GECs is high in proestrus (E 5 – 10 pg/mL; P 0.2 - 0.4 ng/mL), and oestrus (E 20 – 80 pg/mL, P 1 – 5 ng/mL) but decreasing during early metestrus (E 10 – 25 pg/mL, P 15 – 80 ng/mL) [43]. SCs follow the same scheme but with more pronounced fluctuations as reported before [28]. Compared to the anestrous ER expression in SCs a decline during oestrus and early metestrus but an increase during early proestrus was reported by Dhaliwal *et al.*[44] and Veremirsch *et al*. [28] in the native canine endometrium. Therefore, the reduction of ERs in SCs due to 30pg/mL E (comparable to estrous/early metestrous plasma E levels) appears to be analogous to the *in vivo* situation.

In the monolayer culture system of canine endometrial cells ERs in both cell types increased due to 100 pg/mL E [5]. This reaction of SCs may be a result of separated single cell-type culturing in a 2D cell culture system due to a lack of interactions between stromal and epithelial cells. Haslam *et al.*[20] demonstrated that in the mammary gland oestrogenic effects on glandular epithelial cells are controlled by mammary stromal cells *in vivo* and *in vitro*[45]. Direct supplementation of E to mammary epithelial cells cultured alone did not show any effects, neither morphological nor pro-proliferative, although ERs were expressed in these epithelial cells [46]. Pierro *et al.*[41] were able to induce an E dose-dependent increase in proliferative activity in human endometrial epithelial cells only when these were co-cultured with endometrial stromal cells, underlining the importance of epithelial-stromal signaling in hormonal transduction.

PR expression in the GECs was reduced in our 3D cell culture system following E supplementation (100 pg/mL and 30 pg/mL E) but increased with 15 pg/mL E for 48 hr. In the native endometrium PR expression in the GECs is high during early proestrus and oestrus but declines during early and late metestrus compared to anestrous levels [12,44]. PR expression in SCs *in vitro* increased following supplementation of 15 pg/mL and 100 pg/mL E, but decreased after treatment with 30 pg/mL E for 48 hr. PR expression in SCs *in vivo* declines from early to late metestrus compared to anestrous levels, but increases during early proestrus to reach anestrous levels during oestrus [12,44]. The achieved results demonstrate that the two different cell types of the 3D cell culture system partially react comparable to the normal canine endometrial tissue. The increase of PR-positive GECs in the 3D cell culture system upon treatment with 15 pg/mL E is comparable to the increase of PR expression during proestus/early oestrus, and the decline of PR expression in SCs with 30 pg/mL E *in vitro* mirrors the effects during early metestrus *in vivo.* In a human endometrial co-culture system, addition of E induced increased PR-positive immunoreactivity of endometrial epithelial cells [47]. Comparable results were observed in the murine endometrium model of Chung *et al.*[48], demonstrating an increase of PR expression in co-cultured endometrial epithelial cells due to E supplementation. In both studies, endometrial epithelial cells were separated by membranes (inserts) from the stromal cells. In contrast to our 3D organotypic endometrial model neither physiological glandular architecture nor contact between stromal and epithelial cells was provided. Bläuer *et al.*[47] used epithelial organoids embedded in Matrigel™, which formed glandular structures within 24 hr, to show the positive response of epithelial PRs to E supplementation. The decrease of PRs in our studies after treatment with 100 pg/mL and 30 pg/mL E in GECs and 30 pg/mL E in SCs, in contrast to the positive regulation of PRs in GECs and SCs following 15 pg/mL E after 48h culturing period, may reflect the importance of tissue composition (cell-cell contacts) on the one hand, and may display canine-specific reactions of GECs and SCs to the supplemented hormones on the other. Interpretation of species-specific reactions to supplemented steroid hormones in co-culture or 3D-culture systems has to be carried out carefully because of the different culture techniques and ECM compositions.

Another important fact concerning endometrial ER and PR expression patterns in GECs and SCs in the native canine endometrium is that, especially for PRs, different regions of the glands have to be considered due to the different expression patterns of PRs. Vermeirsch *et al.*[12] described distinct PR expression of the basal portion of endometrial glands and their glandular ducts reaching to the surface epithelium. Due to our extraction technique that obtains intact endometrial glands, these regions are no longer distinguishable in the histological sections, and furthermore we do not know if the loss of contact to the surface epithelium induces additional changes of PR (and ER) expression patterns. Nevertheless, we are convinced that working with isolated primary endometrial glands more closely resembles the physiological situation than working with secondary constructed spheroids [10].

The effects of P in the 3D cell-culture system were demonstrated in a decline of ERs in GECs and SCs due to supplementation of 15 ng/mL P for 48 hr. Supplementation of 3 ng/mL P for 48 hr led to a reduction of ER-positive SCs, whereas GECs showed a minimal increase of ER expression after 48 hr in culture. Different authors [13,28] reported that ER expression patterns in the native canine endometrium showed declining values due to increasing serum P levels during oestrus. Increasing ER expression accompanied *in vivo* decreasing serum P values (below 1 ng/mL) during late metestrus were observed in GECs after treatment with 3 ng/mL P. In contrast, in the 2D cell culture system of Galabova *et al.*[5] ER expression in co-cultured GECs was reduced following 3 ng/mL P. In the human endometrial cell culture model of Classen Linke *et al.*[49] endometrial epithelial and stromal cells showed significant down-regulation of ER and PR expression following gestagen supplementation. Similarly Bläuer *et al.*[47] demonstrated reduced PR expression in epithelial organoids after medroxyprogesterone acetate supplementation, to an undetectable level. PR expression in our 3D-cultured SCs increased after 24 hr supplementation with 30 ng/mL P (compared to the hormone free cultivated control group), comparable to the increase of PR expression in SCs at the end of oestrus or early metestrus. These discrepancies with other endometrial epithelial cell cultures may be explained by species-specific reactions of endometrial GECs and SCs, corroborated by the fact that the prolonged serum P levels in the bitch during metestrous are unique to canids [43,50].

### Proliferative activity of endometrial glands and stromal cells in the 3D cell culture system show minor reactions to the added steroid hormones

As the proliferative activity of GECs and SCs in the standard medium was below 1% and of GECs and SCs in the hormone-free medium below 0.2% after a 48-hr culture period, high SD values were inevitably produced from the statistical analysis. In the basal glands of the native canine endometrium, proliferative activity is at low levels in proestrus, with values rapidly increasing in oestrus and early metestrus, followed by declining values in late metestrus and anestrus [15]. Neither in the canine monolayer endometrial culture system [5] nor in our 3D cell-culture system were clear effects of E on proliferative activity observed.

However, supplementation of 30 ng/mL P for 24 hr induced a significant rise in proliferative activity in GECs and SCs in the 3D cell-culture system, compared to the hormone-free cultured cells. Comparable effects of low doses of P were observed in the 2D cell-culture system of Galabova *et al.*[5]. In the native canine endometrial tissue, proliferative activity of the stromal cells is highest in proestrus under oestrogenic influence, and decreases during oestrus with increasing plasma P levels [15]. These divergent reactions of the cells to the steroid hormones *in vitro* concerning proliferative activity compared to the *in vivo* situation may be a result of the single use of either E or P. For example, in the murine endometrium model of Chang *et al.*[48] P supplementation alone did not show any effects on the proliferative activity of SCs, but in combination with E it had inhibitory effects on E-induced proliferative activity. It has to be considered that in the (canine) endometrium, different cell populations (surface and glandular epithelial cells, epithelial cells of the crypts as well as stromal cells and endothelial cells) in different regions (surface, basal region) show different patterns of proliferative activity during the individual cyclic phases. Van Cruchten *et al.*[15] described increased mitotic activity of the surface epithelium, the stroma, the blood vessels and the crypts during proestrus, whereas for the basal glands, proliferative activity increased during oestrus compared to late metestrus and anestrus. In the basal endometrial glands a positive correlation of serum P levels and proliferative activity were observed, whereas in the other cell groups this activity positively correlated with levels of E. Van Cruchten *et al.*[15] concluded that regulation of the proliferation in the canine endometrial surface epithelium, the stroma, the blood vessels and the crypts is different from that in the basal glands. These results highlight the problems faced by researchers isolating, and subsequently culturing and analyzing, isolated endometrial glands and stromal cells originating from the different endometrial regions.

## Conclusions

We have been able to demonstrate the advantages of a 3D cell-culture model of the canine endometrium over monolayer cultures, for experimental approaches due to the differentiated and polarized cell conditions. We found pronounced effects due to single steroid hormone supplementation on ER and PR expression in epithelial and stromal cells. However, in the bitch the prolonged P phase during metestrous as well as the importance of the E:P ratio for the sensitive balance of steroid hormone receptor expression are unique scenarios, and therefore the mimicry of this special hormonal situation in the cycling canine endometrium deserves particular consideration in further studies.

## Abbreviations

3D: Three dimensional; DAPI: 4’,6-Diamidin-2-phenylindol; DPBS-AB: Dulbecco’s Phosphate Buffered Saline containing antibiotics; E: oestrogen (17β-estradiol); ECM: Extracellular matrix; ER: Oestrogen receptor; FCS: Fetal calf serum; GEC: Glandular epithelial cell; HPA: Helix Pomatia Agglutinin; P: Progesterone; PR: Progesterone receptor; SC: Stromal cell; SD: Standard deviations; TEM: Transmission electron microscopy; UEA: Ulex Europaeus Agglutinin; WGA: Wheat Germ Agglutinin.

## Competing interests

The authors declare that they have no competing interests.

## Authors’ contributions

CB conceived the study, performed cell culture experiments, histological, histochemical and immunohistochemical as well as transmission electron microscopy evaluation, and drafted the manuscript. TA participated in the design of the study regarding statistical analyses, and helped to draft the statistical part of the manuscript. SS provided the tissue samples, prepared clinical anamneses and cycle determination of the animals, and revised the manuscript. CA participated in the study design and revised the manuscript. IW conceived the study, participated in the study design and coordination, and helped to draft the manuscript. All authors read and approved the final manuscript.

## Supplementary Material

Additional file 1: Table S1 Detailed scoring results for expression of estrogen and progesterone receptors and proliferative activity in glandular epithelial cells (GECs) and stromal cells (SCs) during different culturing media (standard and hormone free medium as well as supplemented estrogen E and progesterone P in different dosages for 24 and 48 hours, respectively); all values are listed as percentage values ±SD. Click here for file
