# Supplementary material for: Effects of steroid hormones on differentiated glandular epithelial and stromal cells in a three dimensional cell culture model of the canine endometrium
Source: BMC Vet Res. 2013 Apr 24;9:86. doi: 10.1186/1746-6148-9-86 (PMC3660264; doi:10.1186/1746-6148-9-86)
Supplement: Additional file 1: Table S1 — Detailed scoring results for expression of estrogen and progesterone receptors and proliferative activity in glandular epithelial cells (GECs) and stromal cells (SCs) during different culturing media (standard and hormone free medium as well as supplemented estrogen E and progesterone P in different dosages for 24 and 48 hours, respectively); all values are listed as percentage values ±SD. [file 1746-6148-9-86-S1.pdf]

Cell culture medium / time	Immunohistochemical evaluation / scoring					
	Estrogen receptors		Progesterone receptors		Proliferative activity	
	GECs	SCs	GECs	SCs	GECs	SCs
standard / 24h	96,23 ± 3,05	71,07 ± 11,07	61,21 ± 5,00	61,37 ± 4,61	0,03 ± 0,09	0,20 ± 0,16
standard / 48h	86,32 ± 7,03	51,41 ± 22,11	57,42 ± 6,70	52,39 ± 8,07	0,04 ± 0,07	0,18 ± 0,24
hormone free / 24h	69,73 ± 7,12	67,67 ± 11,58	64,65 ± 12,72	46,71 ± 11,31	0,25 ± 0,41	0,06 ± 0,08
hormone free / 48h	45,92 ± 7,78	25,67 ± 9,56	57,71 ± 8,30	38,15 ± 13,12	0,13 ± 0,24	0,16 ± 0,22
E 100 pg/mL / 24h	93,99 ± 4,87	64,02 ± 11,19	74,93 ± 12,48	61,93 ± 7,17	0,05 ± 0,09	0,00 ± 0,00
E 30 pg/mL / 24h	94,61 ± 7,18	58,79 ± 7,31	53,61 ± 5,96	54,34 ± 7,00	1,13 ± 0,60	0,05 ± 0,09
E 15 pg/mL / 24h	99,13 ± 0,15	56,56 ± 5,54	70,45 ± 9,31	52,41 ± 7,66	0,74 ± 0,44	0,30 ± 0,04
E 100 pg/mL / 48h	99,94 ± 0,10	46,16 ± 6,48	69,41 ± 12,13	65,80 ± 5,92	0,03 ± 0,06	0,00 ± 0,00
E 30 pg/mL / 48h	92,00 ± 5,27	16,84 ± 0,33	49,05 ± 7,97	24,16 ± 4,49	0,30 ± 0,29	0,14 ± 0,03
E 15 pg/mL / 48h	98,52 ± 1,92	45,69 ± 1,52	74,36 ± 12,74	60,16 ± 4,17	0,29 ± 0,25	0,38 ± 0,17
P 30 ng/mL / 24h	96,97 ± 3,77	73,86 ± 13,76	55,50 ± 4,77	59,93 ± 6,15	0,56 ± 0,20	0,53 ± 0,09
P 15 ng/mL / 24h	73,52 ± 9,86	60,17 ± 6,83	63,40 ± 3,37	34,20 ± 4,79	0,33 ± 0,29	0,12 ± 0,12
P 3 ng/mL / 24h	95,80 ± 3,48	99,70 ± 0,24	69,48 ± 8,47	70,58 ± 5,87	0,95 ± 0,19	0,29 ± 0,25
P 30 ng/mL / 48h	81,91 ± 9,69	69,94 ± 4,21	59,63 ± 6,13	56,26 ± 1,03	0,90 ± 0,38	0,50 ± 0,13
P 15 ng/mL / 48h	59,00 ± 9,66	16,57 ± 1,90	72,97 ± 2,09	61,91 ± 1,95	0,50 ± 0,62	0,05 ± 0,09
P 3 ng/mL / 48h	99,70 ± 0,24	31,33 ± 2,27	72,77 ± 4,86	56,71 ± 5,72	0,29 ± 0,25	0,44 ± 0,26

Tab.1. supplementary file - Detailed scoring results for expression of estrogen and progesterone receptors and proliferative activity in glandular epithelial cells (GECs) and stromal cells (SCs) during different culturing media (standard and hormone free medium as well as supplemented estrogen E and progesterone P in different dosages for 24 and 48 hours, respectively); all values are listed as percentage values ±SD.
